# Conization Using an Electrosurgical Knife for Cervical Intraepithelial Neoplasia and Microinvasive Carcinoma

**DOI:** 10.1371/journal.pone.0131790

**Published:** 2015-07-08

**Authors:** Libing Xiang, Jiajia Li, Wentao Yang, Xiaoli Xu, Xiaohua Wu, Huaying Wang, Ziting Li, Huijuan Yang

**Affiliations:** 1 Department of Gynecological Oncology, Fudan University Shanghai Cancer Center, Department of Oncology, Shanghai Medical College, Fudan University, Shanghai, 200032, China; 2 Department of Pathology, Fudan University Shanghai Cancer Center, Department of Oncology, Shanghai Medical College, Fudan University, Shanghai, 200032, China; The University of Hong Kong, Queen Mary Hospital, HONG KONG

## Abstract

**Objective:**

The aim of the present study was to evaluate the incidences of margin involvement, disease relapse, and complications in patients who had undergone conization using an electrosurgical knife (EKC) for cervical intraepithelial neoplasia (CIN) or microinvasive carcinomas (micro-CAs).

**Materials and Methods:**

A retrospective case series analysis was performed with a total of 1359 patients who underwent EKC in Fudan University Shanghai Cancer Center between June 2004 and July 2010.

**Results:**

The median age of the patients was 39 years old (range: 19-72). Conization revealed the presence of CIN in 1113 (81.9%) patients, micro-CA in 72 (5.3%) patients and invasive carcinomas in 44 (3.2%) patients. The remaining 130 (9.6%) patients were free of diseases in the cone specimens. Positive surgical margins, or endocervical curettages (ECCs) were found in 90 (7.6%) patients with CINs or micro-CAs. Three factors were associated with positive margins and ECCs and included age (>50 years; odds ratio (OR), 3.0, P<0.01), postmenopausal status (OR, 3.1, P<0.01) and microinvasive disease (OR, 2.7, P<0.01). One thousand and eighty-nine (92.0%) patients were followed-up regularly for a median follow-up duration of 46 months (range: 24-106 months). Disease relapse was documented in 50 (4.6%) patients. Eighty-two (6.0%) cases experienced surgical complications that needed to be addressed, including early or late hemorrhages, infections, cervical stenosis, etc.

**Conclusions:**

Our patients demonstrated that EKC was an alternative technique for diagnosis and treatment of CIN or micro-CAs with relatively low rate of recurrence and acceptable rate of complications. A randomized clinical trial is warranted to compare EKC, CKC and LEEP in the management of CIN or micro-CA.

## Introduction

Cervical intraepithelial neoplasia (CIN) is a precancerous disease that precedes invasive cervical carcinoma. The prevalences of CIN in the Chinese population are 3.1% for CIN1, 1.5% for CIN 2 and 1.2% for CIN3[1]. Given the large population of China, the burden of this disease is heavy. The early detection and appropriate management of CIN is an efficient strategy to prevent preinvasive diseases from progressing to invasive diseases. Conization is a standard surgical procedure for the treatment of high-grade CIN and is also a conservative treatment for microinvasive carcinoma (FIGO stage 1A1, micro-CA) in patients who desire to maintain their fertility. Currently, the loop electrosurgical excision procedure (LEEP) is more commonly used in clinical practice in China than other techniques, such as cold-knife conization (CKC) or laser conization. However, margin involvements are more frequently observed in LEEP than in CKC conization and might lead to greater recurrence and further treatments[2–5].

In the Department of Gynecologic Oncology of Fudan University Shanghai Cancer Center in Shanghai, China, conization using a electrosurgical knife (EKC) has been routinely used for the treatment of high-grade CINs and micro-CAs since September 2003. The technique was shown to be an easily accomplished technique that requires a simple setup and results in good hemostasis in our short-term observation study[6, 7]. However, this surgical procedure has not been well accepted due to margin carbonization and limited experience. In the present study, we present our seven-year experience with EKC for CINs and micro-CAs and long-term follow-up data.

## Materials and Methods

### Patients

This is a retrospective case series study of consecutive women who underwent EKC in the Fudan University Shanghai Cancer Center (FUSCC) from September 2003 to July 2010. This study was reviewed and approved by the Ethics Committee of FUSCC and was exempt from written informed consent requirements (IRB 090876/6). The indications for conization were pathologically proven high-grade CIN (HG-CIN), stage 1A1 microinvasive cervical carcinoma, carcinoma in situ with suspected invasion, glandular dysplasia, residual HG-CIN after LEEP and persistent low-grade CIN (LG-CIN).

### Conization

The procedure of EKC was just like CKC, in which a cold knife was replaced by an traditional monopolar electrosurgical knife. To reduce thermal damage to the specimens, attention was given to avoiding prolonged contact between the knife blades and the specimens. The extents of the excisions were adjusted according to the grade and extent of the disease, although 3- to 5-mm ectocervical and endocervical resection margins were typically used. The cone height represents the length of the tissue removed from the endocervical canal and was dependent on the deep endocervical extensions of the lesion, the patient’s desires regarding childbearing, and the visibility of the transformed region. However, a cone height of at least 10mm was required for each patient, except for young patients desiring childbearing. Endocervical curettage (ECC) was performed to exclude the presence of residual lesions if the transformed zone was not visible or was only partially visible, and was emphasized in patients older than 40 years.

### Pathological assessment

The cone specimens were cut apart from the anterior lips at the 12 o’clock position, fixed in 10% formalin, and embedded in paraffin. Twelve pieces of 4-μm serial sections were stained with hematoxylin-eosin (HE) and examined. Histopathologic assessments of each section were carefully performed and reported. Surgical margins (ectocervical, endocervical, and deep stromal) were defined to be positive based on the presence of CIN or invasive carcinoma at the edge of the specimens.

### Follow-up

The first post-treatment follow-up visit was scheduled for three months after conization, and follow-up were scheduled at six-month interval for two years and once a year for at least three years thereafter. The patients without significant findings during this period were returned to annual cervical cytology in the community. In cases of micro-CA, a strict follow-up involving visits at three-month interval for the first two years and six-month intervals for the subsequent three years was initiated. The complications that needed to be addressed were recorded at the follow-up visits and included as peri- and post-operative hemorrhage, persistent vaginal discharge, abdominal pain, pelvic pain, infections, amenorrhea due to cervical stenosis, etc. Liquid-based cytology was performed for each patient at each visit. In some patients, high-risk types of human papillomavirus were detected with the techniques of hybrid capture 2 or genotyping. The indications for colposcopy-directed biopsies were based on the presence of abnormal cytology at any visit or hr-HPV-positive statuses in two consecutive visits.

### Statistical analyses

The clinicopathological data were retrieved prospectively by Jiajia Li from the medical records and included the patient's age, menopausal status, gravidity, parity, symptoms including vaginal bleeding or discharge, and signs including the extent of cervical erosion, the severity of the final diagnosis, surgical margin involvement, and pathological results from the ECC. These data and the follow-up data were prospectively entered into an SPSS database and updated during the follow-up visits. The end of the observation period was Mar 31, 2013. The data were analyzed anonymously by Libing Xiang, Huijuan Yang in this study. Fisher's exact tests were employed to identify the risk factors that were associated with margin involvement. Statistical significance was defined as P<0.05. All statistical analyses were performed using SPSS 16.0 (SPSS Inc., Chicago, IL, USA).

## Results

A total of 1359 patients were included in this retrospective case series study. The median age was 39 years old (range:19 ~72 years). Among the patients, 93.3% were pre-menopausal, and 6.7% were post-menopausal, 92.6% had children.

Majority of the patients (92.2%) underwent conization with therapeutic intentions, and 7.8% of the patients underwent conization with diagnostic intentions. In total, conization revealed the presence of CIN in 1113 (81.9%) patients, and 116 (8.5%) patients were confirmed to have invasive carcinomas that included 72 FIGO stage 1A1 carcinomas (micro-CA), 29 FIGO stage 1A2 carcinomas, and 15 FIGO stage Ib1 carcinomas(S1 Fig). The remaining 130 (9.6%) patients were found to be free of diseases in the cone specimens. Table 1 compares the histopathologies of the colposcopy-directed biopsies and those of the cone specimens. The histopathologies were consistent in 1000 (76.7%) patients, and 109 (8.4%) patients had more severe lesions in the cone specimens.

**Table 1:** Comparison between the histopathologies of cone specimens and those of colposcopic-directed biopsies.

Punch biopies	Cone biopies				Sum (%)
	NC ^a^ (%)	LG-CIN(%)	HG-CIN (%)	ICC(%)	
LG-CIN	18(36.7)	21(42.9)	10(20.4)	0(0)	49(3.8)
HG-CIN	81(7.1)	78(6.8)	906(79.3)	77(6.7)	1142(87.6)
CIS with Suspected ICC	6(6.6)	3(3.3)	60(65.9)	22(24.2)	91(7.0)
Microinvasive ICC	1(4.8)	0(0)	7(33.3)	13(61.9)	21(1.6)
Sum (%)	106(8.1)	102(7.8)	983(75.4)	112(8.6)	1303(100)

^a^ NC: normal cervix, ICC: invasive cervical carcinoma, LG-CIN: low grade cervical intraepithelial neoplasia, HG-CIN: high grade cervical intraepithelial neoplasia, CIS: carcinoma in situ

Among the 1113 patients with CINs and the 72 patients with micro-CAs whose diseases could be definitively treated with conization, positive surgical margins or endocervical curettages (ECC) were found in 90 (7.6%) patients, including 47 (4.0%) with positive margins, 34 (2.9%) with positive ECC and nine (0.8%) with both. Table 2 presents the risk factors associated with margin involvement. The patients’ age, menopausal status and disease severity were found to be associated with the involvement of the surgical margin and ECC. The odds ratios were 3.0 for age ≥50 years (95% confidence interval (95% CI): 1.6–5.6, P<0.01), 3.1 for post-menopausal status (95% CI: 1.6–5.9, P<0.01) and 2.7 for micro-CAs (95% CI: 1.4–5.1, P<0.01).

**Table 2:** Clinicopathological parameters associated with positive conization margins or ECCs in 1185 patients with CINs or micro-CAs ^a^.

Parameters	Cases	Margin/ECC		OR (95%CI)	P value ^b^
		Negative	Positive		
Age (years)				3.0(1.6–5.6)	<0.01
<50	1108	1032	76		
≥50	77	63	14		
Menopausal status				3.1(1.6–5.9)	<0.01
Premenopausal	1115	1038	77		
Postmenopausal	70	57	13		
Parity				0.5(0.1–1.5)	0.27
Nulliparous	81	78	3		
Parous	1104	1017	87		
Disease severity				2.7(1.4–5.1)	<0.01
CIN	1113	1035	78		
Micro-CA	72	60	12		

^a^ One hundred seventy-four patients were no counted, including 44 patients with stage 1A2+ carcinoma and 130 patients without diseases in cone specimens.

^b^ Fisher’s exact test was used.

CIN: cervical intraepithelial neoplasia; micro-CA: microinvasive carcinoma; ECC: endocervical curettage; OR: odds ratio

**Fig 1:**
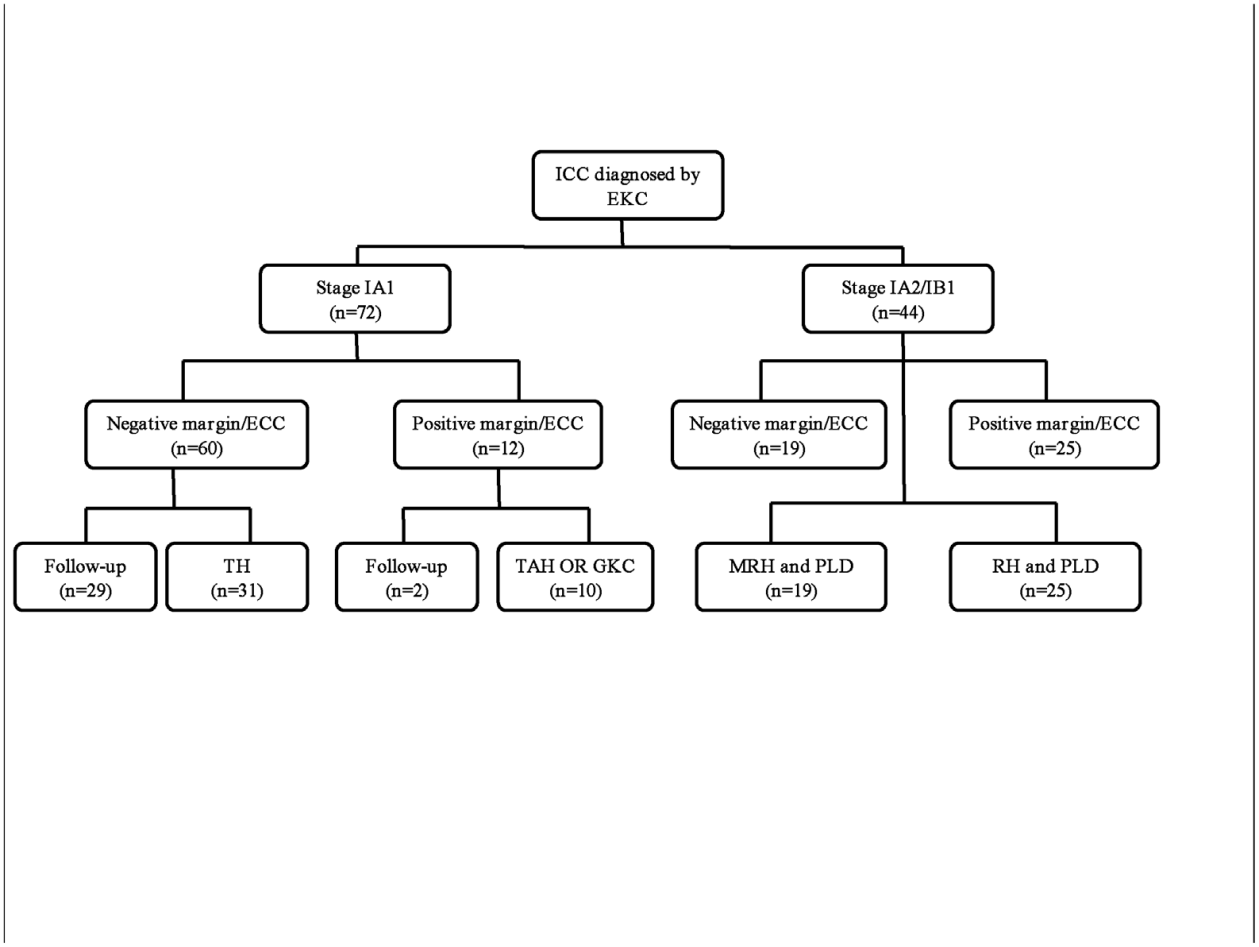
Management after conization in the 116 patients with invasive disease (ICC: invasive cervical carcinoma; EKC: conization using a traditional monopolar electrosurgical knife; ECC: endocervical curettages (ECC); TH: total hysterectomy; MRH: modified radical hysterectomy; RH: Radical hysterectomy; PLD: pelvic lymphadnectomy).

For the further management after conization, all the 44 patients with FIGO stage 1A2 or 1B1 carcinomas underwent radical hysterectomy or trachelectomy and pelvic lymphadenectomy. Among the patients with micro-CAs, 10 patients with positive margins or ECCs underwent subsequent hysterectomy or secondary conization; 31 patients without margin involvement underwent hysterectomy because they did not desire to maintain their fertility or due to concurrent uterine myomas or adenomyosis; the other 31 patients were followed up closely (2 cases with margin involvement and 29 cases without margin involvement). (Fig 1) Among the CIN patients, 43 (55.1%) of the 78 patients with positive margins or ECCs underwent subsequent hysterectomy or secondary conization; whereas, 44 (4.3%) of 1035 patients without margin involvement underwent hysterectomy because they did not desire to maintain their fertility or due to uterine myomas or adenomyosis; the rest 1026 patients were followed up. For those CIN patients and stage 1A1 patients who underwent secondary surgeries, residual CIN 2+ lesions were more commonly observed in the secondary specimens of the patients with margin or ECC involvement (24 /53,45.3%) compared to the patients without involvement (17/78, 21.8%) (*P*<0.01). Thus, positive surgical margins and ECCs represented incomplete conization and were confirmed to be associated with residual diseases in the remaining cervix after repeat conization or hysterectomy.

Among the 1184 (87.1%) women who successfully preserved their uteruses after conization, 1089 (92.0%) of the patients were followed-up regularly according to our protocol. During the median follow-up duration of 46 months (range:24–106 months), cervical neoplastic lesions was documented in 50(4.6%)patients. Among these patients, 23 recurred with LG-CIN, 21 with HG-CIN, two with HG-CIN and concurrent vaginal intraepithelial neoplasia (VaIN), and four cases progressed to invasive carcinoma. The median interval between conization to the documentation of disease relapse was 10 months (range: 3 to 64). Twenty-eight (56.0%) patients relapsed within one year of conization, and 37 (74.0%) patients relapsed within two years of conization.

A total of 82(6.0%)patients suffered from surgical complications that needed to be addressed (Table 3). Seventeen (1.3%) patients were admitted to the hospital for management of complications, including 12 patients who were admitted for wound hemorrhage from the cervix within one month of conization, four patients for cervical stenosis and one patient for deep vein thrombosis (DVT). All bleeding was successfully treated with local compression, suturing, or cauterization. Dilatation of the cervical canal was performed in the four patients with hematometra due to cervical stenosis after conization. Another 65 (4.8%) patients experienced conization-related symptoms and signs that influenced their daily lives and needed to be addressed, such as persistent vaginal discharge, chronic pelvic pain, cervical polyps, cervical ectropion, etc.

**Table 3:** Conization-related complications needed to be addressed.

Complications	Cases (%)
Complications needed to addressed in hospital	17 (1.3%)
Hemorrhage of the wound	12
Cervical stenosis	4
Deep vein thrombosis	1
Conization-related symptoms or signs that influenced patients' daily lives	65(4.8%)
Persistent vaginal bleeding	17
Chronic pelvic pain	15
Persistent vaginal discharge	10
Cervical polyps	10
Cervical ectropion	7
Other complaints	6

## Discussion

Conization of the cervix is a standard conservative treatment for patients with HG-CIN to prevent progression to invasive cancer. Conization is also an efficient diagnostic tool for suspicious squamous and glandular dysplasias that arise from the cervix. The techniques for therapeutic and diagnostic conization are identical. Conization can be performed with a scalpel (cold-knife conization, CKC), an electrosurgical loop (loop electrosurgical excision procedure, LEEP), or a laser, and these methods have different advantages and disadvantages. LEEP has been widely used worldwide due to its convenience, low blood loss, quality preservation of the margins for histological evaluation and low incidence of postoperative hemorrhage. However, margin involvement is common with the LEEP technique, and approximately half of patients undergo LEEP in multiple passes[8–10]. Conization with EKC resembles CKC but overcomes the drawbacks of CKC in terms of hemostasis. Traditional monopolar electrosurgical knives exist in every operating room, even in low-resource clinical settings, and the manipulations are easily performed without special instruments. The short-term efficacy and safety of EKC were reported in our previous study[6, 7]. In the present study, we evaluated long-term evidence regarding margin involvement, disease relapse, and complications in patients who had undergone this procedure.

Margin involvement in the cone specimens following LEEP or CKC might be indicative of the probability of residual disease in the remaining cervix and influences decisions regarding follow-up and secondary surgeries. Margin involvement has been recognized as one of the most important predictors of disease recurrence and progression[10–13]. Thus, margin involvement is also the most important concern in the EKC procedure. In the present study, positive surgical margins and ECCs were found in 4.7% and 3.6% of the patients, respectively. Both of these levels are lower than those reported in other studies[2, 10, 11, 14]. These lower prevalences might be explained by the similarity of the EKC and CKC techniques. According to the literature, CKC more consistently produces negative margins, particularly endocervical margins, than does LEEP[2, 3, 15]. This finding might be attributable to the fact that CKC removes the lesions more deeply than does LEEP. It has been confirmed that a conization depth less than 10 mm might be a risk factor that predicts endocervical resection margins[16, 17]. Moreover, Papoutsis found that a cone height less than 10mm was the best predictor of margin involvement and had the highest sensitivity (100%) among other factors that included cone volume and cone base surface[16]. Although the detailed dimensions of the cone specimens were not examined in the present study, it should be emphasized that our EKC procedure should involve cone depths greater than 10 mm mentioned as in Materials and Methods.

In addition to the technique itself, margin involvement was found to be associated with the patient's age and menopause status in the present study. Some studies have confirmed the relationship between age and endocervical margin but not the relationship between age and ectosurgical margin[18,19]. Bae et al. reported that the risk of endocervical margin involvement increases with the age (the odd ratios were 1.59 for 40– to 59-year-old patients and 4.16 in patients over 60 years compared to patients younger than 40 years) [18]. This finding might be due to the migration of the transformation zone to the cervical canal with the age and following menopause, which leads to difficulties in removing the intact transformation zone in elderly and postmenopausal women. Thus, ECC should be performed to exclude disease residues in the cervical canal in elderly and menopausal women. Subsequent hysterectomies are typically considered for elderly patients with positive endocervical margins or positive ECCs. Conization could be a definitive therapy for patients with microinvasive carcinomas (FIGO stage 1A1) who want to preserve their fertility. However, these patients have more positive margins and ECCs than the patients with high-grade CINs[19]. It was confirmed in the present study (16.7% vs 7.0%, P<0.01). Given that margin status indicative of the possibility of residual diseases and the risks of recurrence and progression, 10 (85.7%) of the 12 patients with margin involvement underwent subsequent surgeries. Decisions regarding simple or radical trachelectomy and simple or radical hysterectomy are based on the patient's desire to maintain her fertility and the severities of the dysplastic cells in the surgical margins and endocervical curettages. Subsequent hysterectomies were also performed in 31 (51.7%) of the patients without margin or ECC involvement as radical treatments.

For patients who preserve their uteruses after conization, regular follow-up visits are required to facilitate the early identification of disease relapse. In this series of 1089 cases, disease relapse was documented in 4.6% of the patients during the observation period. The prevalence of disease relapse in this series was relatively low compared to those reported in the literature, which are extremely variable (from 2% to 43%;median: 9%)[11, 13, 20–22]. The reduced incidence of margin involvement associated with the technique contributed to the low incidence of disease relapse. Moreover, the use of diathermy for hemostasis at the surface of the remaining cervix might be effective for eradicating any remaining dysplastic cells. Additionally, women who had undergone reflex hysterectomies soon after conization due to positive margins or ECCs were not included in this study. Consequently, disease relapse might have been underestimated by examination of only the remaining patients. Four clinicopathological parameters have been identified as independent predictors of recurrence: family history of virus-associated malignancies (odds ratio (OR), 11.0, P<0.001), endocervical curettage (ECC; OR, 4.8, P = 0.013), micro-CA (OR, 4.2, P = 0.021), and surgical margins (OR, 3.4, P = 0.033). Another manuscript on these findings is being written for publication.

The most common conization-related complications were early and late hemorrhages, infections, and cervical stenosis. In the present EKC technique, coagulation of the cone beds is routinely performed after the removal of the cone; thus, peri-operative bleeding is uncommon. However, late bleeding is common during decrustation. In a prospective study, Dane et al. reported that cerclage sutures are more efficient than cauterization in the prevention of early and late hemorrhage[23]. Recently, we employed No. 1 Vicryl polyglactin910 sutures (Ethicon Intl., Johnson & Johnson,Somerville, New Jersey, USA) to suture the remaining cervix circumferentially after conization. Our preliminary observations indicate that this technique resulted in a decrease in the occurrence of late hemorrhages and aided the restoration of the remaining cervix. Cervical stenosis is a particularly important complication that can lead to dysmenorrhea, amenorrhea, and infertility. Cervical stenosis also leads increases in false-negative cytologies and unsatisfactory colposcopies during follow-ups after conization. The incidence of cervical stenosis varies across studies from 1 to 15% depending on the definition employed[24–27]. In the present study, only patients with hematometra and secondary amenorrhea due to conization were considered to have cervical stenosis. Patients with secondary dysmenorrhea were not included in this group. We also lacked the colposcopic evidence to confirm the partial or complete obstetrics of the cervical canals. Thus, the prevalence of cervical stenosis might have been underestimated. Another limitation of this study is that we lacked data regarding secondary infertility and obstetric outcomes due to the limited number of cases who desired to maintain their fertility and the limited follow-up duration. Further observations of greater numbers of cases are needed to reveal the influences of the EKC procedure on infertility and obstetric outcomes.

In this study, 78 patients with negative cone margins underwent hysterectomy, and 17 (21.8%) patients had CIN2+ in the residual cervix. The rate was similar to the study by Park et al.[28]. In their study, 49 CIN or IA1 patients with clear margin underwent subsequent hysterectomy after conization and 10 patients (20.4%) were found to have residual diseases. Inconsecutive sections of histological examination and multifocal lesions of CIN or microinvasive diseases may explain these phenomena. In addition, selection bias and sampling errors were the other reasons. Selection bias did exist in this cohort, as a considerable proportion of patients (13 patients) had CIN lesions near the margin (within 1 mm) and another considerable proportion of patients (31 patients) had micro-CAs.

In conclusion, our patients demonstrated that EKC was an alternative technique for diagnosis and treatment of CIN or micro-CAs with relatively low rate of recurrence and acceptable rate of complications. A further randomized clinical trial is warranted to compare the treatment outcome and obstetric outcome of EKC, CKC and LEEP in the management of CIN or micro-CA.

## Supporting Information

S1 Fig Treatment and follow-up of the 1359 patients with EKC. (JPG)
