# Supplementary figures and images for: Conization Using an Electrosurgical Knife for Cervical Intraepithelial Neoplasia and Microinvasive Carcinoma
Source: PLoS One. 2015 Jul 8;10(7):e0131790. doi: 10.1371/journal.pone.0131790 (PMC4496038; doi:10.1371/journal.pone.0131790)

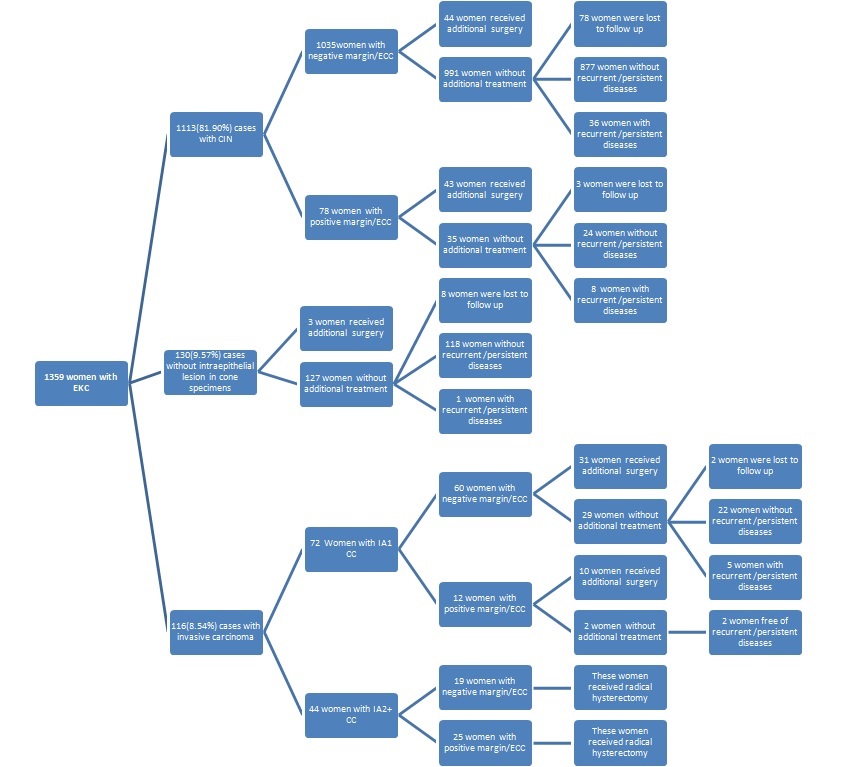

Supplement: S1 Fig — (JPG) [file pone.0131790.s001.jpg]
